# The Effect of L-Theanine Incorporated in a Functional Food Product (Mango Sorbet) on Physiological Responses in Healthy Males: A Pilot Randomised Controlled Trial

**DOI:** 10.3390/foods9030371

**Published:** 2020-03-23

**Authors:** Jackson Williams, Andrew J. McKune, Ekavi N. Georgousopoulou, Jane Kellett, Nathan M. D’Cunha, Domenico Sergi, Duane Mellor, Nenad Naumovski

**Affiliations:** 1 Faculty of Health, University of Canberra, Canberra, ACT 2601, Australia; jackson.williams@canberra.edu.au (J.W.); andrew.mckune@canberra.edu.au (A.J.M.); jane.kellett@canberra.edu.au (J.K.); nathan.dcunha@canberra.edu.au (N.M.D.); 2 Discipline of Biokinetics, Exercise and Leisure Sciences, School of Health Sciences, University of KwaZulu-Natal, Durban, KwaZulu-Natal 4000, South Africa; 3 Research Institute of Sport and Exercise, University of Canberra, Canberra, ACT 2605, Australia; 4 Centre for Health and Medical Research, ACT Health Directorate, Canberra, ACT 2601, Australia; ekavigeorgousopoulou@gmail.com; 5 Medical School, Australian National University, Canberra, ACT 2601, Australia; 6 School of Medicine, University of Notre Dame Australia, Sydney, NSW 2000, Australia; 7 Nutrition & Health Substantiation Group, Nutrition and Health Program, Health and Biosecurity, Commonwealth Scientific and Industrial Research Organisation (CSIRO), Adelaide, SA 5000, Australia; Domenico.Sergi@csiro.au; 8 Aston Medical School, Aston University, Birmingham B47ET, UK; d.mellor@aston.ac.uk

**Keywords:** L-Theanine, amino acid, green tea, bioactive, functional food, blood pressure, heart rate variability, cardiometabolic effect

## Abstract

Consumption of L-Theanine (L-THE) has been associated with a sensation of relaxation, as well as a reduction of stress. However, these physiological responses have yet to be elucidated in humans where L-THE is compared alongside food or as a functional ingredient within the food matrix. The aim of this study was to determine the physiological responses of a single intake of a potential functional food product (mango sorbet) containing L-THE (ms-L-THE; 200 mgw/*w*) in comparison to a flavour and colour-matched placebo (ms). Eighteen healthy male participants were recruited in this randomised, double-blind, placebo-controlled trial. The participants were required to consume ms-L-THE or placebo and their blood pressure (BP) (systolic and diastolic), heart rate (HR), and heart rate variability (HRV) were monitored continuously over 90 minutes. Eleven males (age 27.7 ± 10.8 years) completed the study. Changes in area under the curve for systolic and diastolic blood pressure and HRV over the 90 minute observation period indicated no differences between the three conditions (all *p* > 0.05) or within individual groups (all *p >* 0.05). The values for heart rate were also not different in the placebo group (*p* = 0.996) and treatment group (*p* = 0.066), while there was a difference seen at the baseline (*p* = 0.003). Based on the findings of this study, L-THE incorporated in a food matrix (mango sorbet) demonstrated no reduction in BP or HR and showed no significant parasympathetic interaction as determined by HRV high-frequency band and low-frequency/high-frequency ratio. Further studies should be focussed towards the comparison of pure L-THE and incorporation within the food matrix to warrant recommendations of L-THE alongside food consumption.

## 1. Introduction

Stress is a dynamic condition that acts as a stimulus, affecting arousal factors of individuals in response to challenges and aversive situations. The consequences of stress are strongly influenced by how an individual perceives and appraises the situation in addition to the intensity of the event that triggers the stress response [1,2,3]. Traditionally, consumption of tea is associated with relaxation [3], beneficial health outcomes [4], and an increase in longevity, which is proposed to be due to a number of different bioactive constituents [5,6]. Prior to its consumption, tea leaves undergo processing and based on the type of processing, predominantly leads to seven different types of tea; ‘green’ (unfermented), ‘yellow’, ‘white’, ‘oolong’ (partially fermented), ‘black’ (completely fermented), ‘aged pu-erh’ (drastically fermented and aged), and ‘ripened pu-erh’ tea [7]. The type of processing also affects the formation of different compounds, including but not limited to the composition and degradation of the molecules found in green tea. In green tea, the steaming (or heating) process inactivates the enzyme polyphenol oxidase, which prevents the further fermentation of tea leaves, consequently producing a stable and dry product. During this process, oxidative polymerisation of monomeric flavan-3-ols is inhibited (unlike in the black and other teas), and green tea typically contains higher levels of these flavan-3-ols. Therefore, it can be anticipated that different processing techniques will also influence the levels of other molecules and putative bioactives, such as amino acids.

Derived from the leaves of the *Camellia sinensis* species, green tea is consumed due to its favourable taste and also for its reported therapeutic properties such as cardiovascular benefits, increased relaxation effects and strong, anti-inflammatory [8] and antioxidant effects [6,7,9]. These effects are linked to its many bioactive constituents, including polyphenols [10], particularly catechins [11], and γ-glutamylethylamide, commonly referred to as L-Theanine (L-THE), which is the most abundant and unique non-proteinogenic amino acid in green tea.

The consumption of L-THE is associated with a sensation of relaxation, and is proposed to elicit acute stress-reducing effects [3] as well as providing the characteristic *‘umami’* flavour [6]. In addition, the consumption of L-THE is associated with antihypertensive [12,13], anti-inflammatory properties [8] as well as improvements in cognitive functioning in healthy adults [14] and when consumed in combination with caffeine [10]. Studies report stress lowering effects, expressed physiologically in the form of reductions in heart rate (HR), observed post-completion of a stress-inducing mental arithmetic task after the consumption of L-THE (200 mg) in its relatively pure form [15]. Furthermore, reductions in salivary cortisol post L-THE consumption indicate the putative involvement of L-THE in hypothalamic-pituitary-adrenal (HPA) axis responses have also been reported [15,16]. 

The autonomic nervous system (ANS), consisting of the sympathetic nervous system (SNS) and parasympathetic nervous systems (PNS) [17,18], are of central importance to the homeostatic stress response. Blood pressure is a physiological biomarker that is highly dependant on catecholaminergic neurons [19]. The HR is also considered to be a vital indicator of the stress response, however, the changes in interval between the highest points (R-R interval) of the heartbeat observed on the electrocardiogram (usually referred to as QRS complex), known as heart rate variability (HRV), is becoming a reliable marker for the responses caused by internal and external stressors. Changes in HRV, representing ANS cardiac regulation, can be directly (*via* ANS innervation of the myocardium) or humorally (catecholamines related hormones) mediated [20]. The high frequency (HF) component of HRV ranges from 0.15‒0.4 Hz, and is based on respiratory sinus arrhythmia and mediated predominantly by PNS activity, whereas low frequency (LF) ranges between 0.04‒0.15 Hz, is mediated by both PNS and SNS [21]. Furthermore, the state of being stressed affects HRV domains such as HF, LF and LF/HF ratios, which are used to understand the activity of the PNS and further as an index of autonomic balance [22].

It is proposed that the ‘healthier’ the ANS, the better an individual will be able to exhibit high allostatic resilience [23]. As such, HRV changes are reported to be a potential marker of the relationship between food consumption and disease [24]. These findings were further supported in studies where certain components of dietary intake are associated with higher HRV, for instance the Mediterranean diet, which consists highly of fish and omega-3 fatty acid consumption, fresh produce, [25] as well as healthy weight maintenance [26] all contribute to the potential increase in HRV. On the other hand, reduced HRV is associated with an increased risk for a range of diseases including but not limited to cardiovascular disease [27] and diabetes [28]. This contributes to the possibility that HRV markers provide a fast and convenient way to measure the potential benefits of health status in response to various dietary interventions.

Regardless of the method of delivery of this non-proteinogenic amino acid, L-THE needs to be easily accessible and bioavailable in concentrations sufficient to produce the desired physiological effects. Human studies in which consumption of pure L-THE was provided in capsule form, indicate the most physiologically relevant doses of L-THE range from 0.05−0.4 g, equivalent to a consumption of 2−15 cups of green tea [6,29,30]. One method to deliver these dosages of L-THE is via the integration of pure L-THE in the food matrix as a potential functional food product. Based on the findings of our previous review [6], we propose that incorporating 200 mg of L-THE in a functional food product will provide a convenient and palatable delivery method of a physiologically relevant dose of L-THE. Therefore, the aim of this study was to determine the physiological responses HR, HRV, and blood pressure (BP) in healthy males following the one-off acute ingestion of a potential functional food product (mango sorbet) containing 200 mg of pure L-THE. 

## 2. Methods and Materials

### 2.1. Participants

Participants were informed about the study protocols, provided written consent, and then screened to determine eligibility for participation. Following previously conducted research using similar design and products [11], inclusion criteria included healthy male participants aged 18−65 years old. Participants were excluded from participating if they consume: any functional foods including stanol or sterol ester-containing margarines; weight loss supplements, or commercial dietary products associated with weight loss; currently had or have had any known active pulmonary, hematologic, hepatic, gastrointestinal, renal, premalignant, malignant illnesses; have diabetes (type 1 and type 2); or any thyroid dysfunction.

### 2.2. Procedure

A randomised, double-blind, placebo-controlled, crossover design was used to determine the acute physiological HR, HRV, and BP effects after consumption of mango sorbet containing L-THE (200 mg). This study was approved by the Human Research Ethics Committee of the University of Canberra (HREC-193–2018) and written informed consent was obtained from all participants.

**Figure 1:**
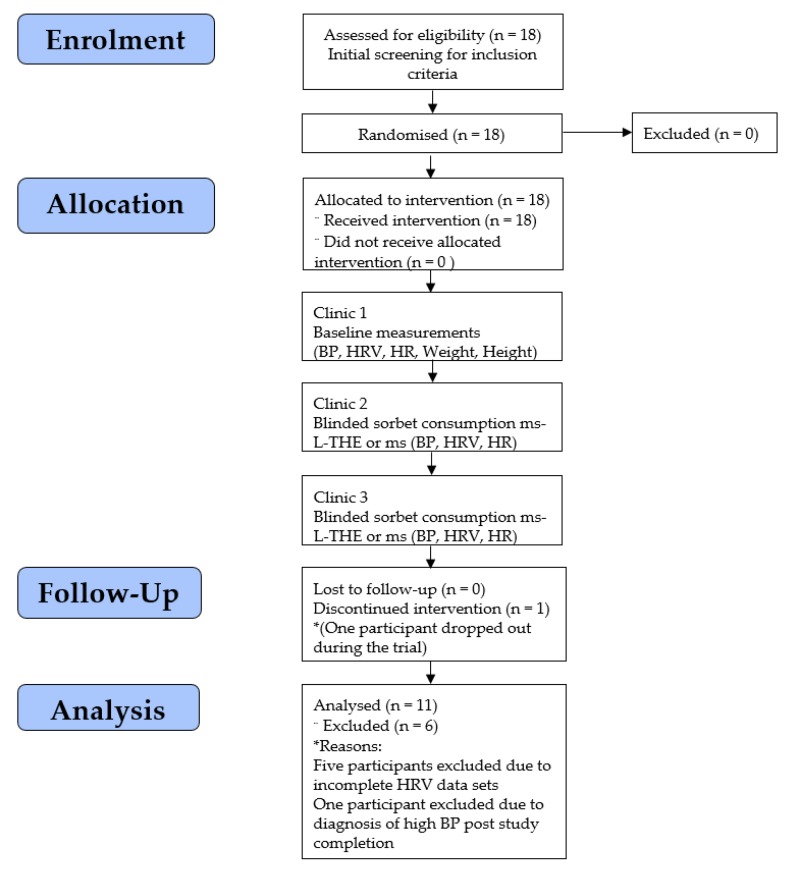
Consort flowchart of clinics attended in the study for the 11 included participants.

Participants were required to attend four clinic visits (Figure 1), initial screening (clinic 1), a baseline measurements clinic (clinic 2) to familiarise participants, and two blinded food consumption clinics (clinics 3 and 4). In the last two clinics, participants consumed the 100 g of the food products mango sorbet, one a placebo without active ingredient (ms) or the treatment mango sorbet containing 200 mg of L-THE (ms-L-THE). The findings from animal studies suggested that L-THE brain levels are increased 30−60 minutes post-consumption. Thus, assessment of the physiological parameters mentioned below occurred immediately post L-THE food product consumption to capture the period and curve at which L-THE exerts its effects.

Allocated randomised treatment sequences were achieved using 4 blocks of random sequencing numbers per intervention (randomizer.org) to ensure the ms and ms-L-THE sorbets administrations were balanced between the participants. The sequence code was placed in a sealed envelope and revealed only at the end of the trial once all participants finalised all of their visits. A 48 h washout period was used between clinics 3 and 4 ensuring the adequate time for the L-THE clearance from the body [29]. Prior to attending clinics 2, 3, and 4, participants were required to fast overnight (at least 8 h) except for water consumption. Participants were also asked to refrain from alcohol for 24 h, and caffeine 12 h prior the commencement of the clinic.

### 2.3. Materials and Reagents

The mango sorbet used in this study was developed based on the modified formulation described elsewhere [11]. Common mangos (Mango pieces, Coles Pty Ltd, Peru/Mexico), caster sugar (CSR, Yarraville, VIC, Australia), were purchased from local commercial suppliers. The L-THE (Suntheanine™, Taiyo Kagaku Co., Ltd, Yokkaichi Japan) was purchased from Ingredient Resources Australia and New Zealand Pty/Ltd (Sydney, NSW, Australia) and was the functional additive (200 mg/100 g *w*/*w*) to the active product. The whey protein concentrate (WPC) was purchased from Professional Whey Pty/Ltd (Erina, NSW, Australia) and added to provide structural stability and rigidity to the food product. The sorbet was selected as the food matrix of choice for this study due to the preference for storage stability of L-THE such as low temperature (less than 4 °C) and stability in an acidic environment (pH range 5−6) [31]. The dosage of L-THE (200 mg) was selected based on the findings of previous studies [29]. The nutritional composition of the ms-L-THE (Table 1), including the macro- and micronutrient data were analysed using the Australian Food Composition Database integrated into the FoodWorks Professional Software (v9, Xyris Software, Brisbane, QLD, Australia).

### 2.4. Outcomes

#### 2.4.1. Blood Pressure

Participants’ blood pressure was determined following the guidelines for the 2nd Australian National Blood Pressure study [32]. Blood pressure was determined using the finger cuff (non-invasive BP nano, AD Instruments, Dunedin, New Zealand), using two inter-inflation cuffs that were placed on the participants in a sedentary position. Continuous readings for systolic, diastolic blood pressure were recorded, as well as inter-beat interval, HR, and mean arterial pressure over 90 minutes. The data were smoothed based on the AD instruments smoothing algorithm to remove outlying data points due to finger movement and further segmented into 18 distinct values from each 5 min interval [11].

#### 2.4.2. Heart Rate Variability

Each participant was provided with a fitness-tracking device heart rate belt (Suunto^®^ T6, Vantaa, Finland) that measures HR and R-R intervals of continuous electrocardiogram QRS heartbeat complexes. Participants were instructed how to use the device, ensuring the belt was placed below the chest muscles across the sternum after water was applied to the undersurface of the belt to ensure conduction [33]. Participants were required to be in a sedentary position for 10 min to establish resting measurements. During each clinic, participants were required to wear the heart rate belt in a sedentary position for the entirety of the clinic. Recordings for all clinics began after 10 minutes of participants being in a sedentary position. However, during consumption clinics, recordings began after complete consumption of each respective sorbet.

### 2.5. Statistical Analysis

All data obtained were analysed as a 5 min average up to 90 min. The obtained R-R interval data were downloaded from the HR monitors via the Suunto T6 Team Management Software (v2.1, Vantaa, Finland) and exported as a text file for time domain and spectral HRV analysis using the Kubios HRV Standard 3.0.2 diagnostic device software (Kuopio, Finland). All R-R interval artefacts were manually corrected using the methodology described by Sookan and McKune (2012). Using total area under the curve (AUC), the non-parametric independent samples Kruskal–Wallis ANOVA was the primary model for analysis for time effects for between-group time relationships the entire 0−90 min block, and further analysed between each 5 min period up to 90 min also using Kruskal–Wallis analysis. Both are presented as medians, 25th and 75th quartiles with Chi-square and *p*-value. For related samples, Friedman’s two-way ANOVA by ranks was applied to test same group relationships for the entirety of 90 min, presented as Chi-square and *p*-value. The HF HRV values were log-transformed prior to analysis. Level of significance was set at α = 0.05. Data were analysed using the SPSS v25 (Armonk, IBM Corp, New York, USA).

## 3. Results

Initially, eighteen healthy male adults were recruited with 17 completing all four study visits. Following the completion of the study, data were analysed from 11 participants (age 27.7 ± 10.8 years, weight 90.6 ± 16.8 kg) due to the following; one participant voluntarily discontinued the trial due to lost contact; data of five participants were excluded due to insufficient data points collected due to equipment malfunction, whilst one participant was excluded post-study due to a clinical diagnosis of high blood pressure. All descriptive statistics for systolic and diastolic BP, heart rate, HF HRV including 1st and 3rd quartiles, median, total AUC values described in Table 2.

### 3.1. Systolic and Diastolic Blood Pressure

**Figure 2:**
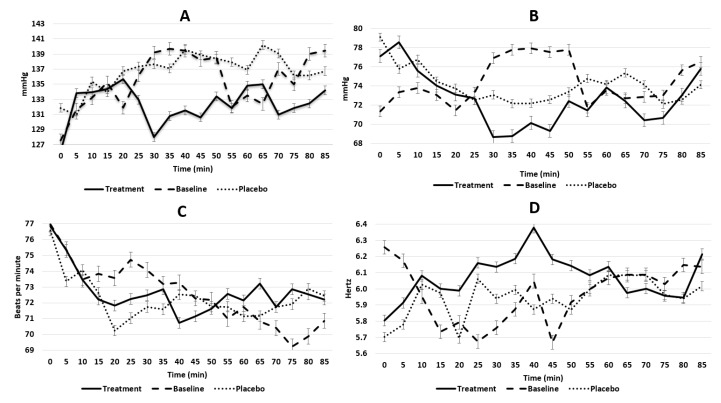
The changes in physiological parameters: systolic blood pressure (**A**); diastolic blood pressure (**B**), heart rate (**C**) and log of high-frequency heart rate variability (**D**) over the 90 minute period for the 11 included particpants.

For systolic BP, between-group analysis for AUC did not demonstrate any significant effects for the entire 90 minute period (Figure 2A) (median; 1323 mmHg, 25th; 1157 mmHg, 75th; 1462 mmHg, *p* = 0.839). Further analysis of the individual 18 × 5 minute time points showed no significant differences between the three clinic visits (*p* > 0.05). The within-group analysis provided all non-significant results respectively for the entirety of the 90 minute groups (all *p* > 0.05).

For diastolic BP, between-group AUC analysis showed no significant differences during the entirety of the 90 minutes (Figure 2B) amongst either of the conditions (median; 1323 mmHg, 25th; 1157 mmHg, 75th; 1462 mmHg, *p* = 0.922). Similarly, between-group analysis of the individual 18 × 5 minute time points also showed no significant differences between each of the three clinic visits (all *p > 0.05*). Same group analysis test indicated no significant differences within each of the conditions (all *p* > 0.05).

### 3.2. Systolic/Diastolic Ratio

The ratio of systolic/diastolic AUC analysis showed no significant differences during the entirety of 90 min between either of the conditions (median; 32.3, 25th; 31.1, 75th; 35.7, *p* = 0.970). Further, analysis of the individual 18 × 5 min time points showed no significant differences between each of the three conditions (all *p* > 0.05). Same condition analysis showed no significant differences within the baseline group (*p* = 0.761), whereas significant differences were observed in the ms-L-THE group (*p* = 0.003) and the ms group (*p* = 0.003). 

### 3.3. Heart Rate

Between-group AUC analysis showed no significant differences during the entire 90 minutes between either of the conditions (median; 1325, 25th; 1222, 75th; 1385, *p* = 0.996). Further analysis of the individual 18 × 5 min time points also showed no significant differences between each of the three conditions (all *p* > 0.05), whereas within-group analysis showed significant differences within the baseline group (*p* = 0.003) as well as within the ms-L-THE group (*p =* 0.066) (Figure 2C). No significant differences were observed in the ms group (*p =* 0.060).

### 3.4. Sympathetic Activity

#### 3.4.1. High-frequency HRV

AUC HF analysis showed no significant time effects between groups (median; 97.3, 25th; 6.59, 75th; 120, *p* = 0.974). Analysis of the individual 18 × 5 min time points also showed no significant differences between each of the three conditions (all *p* > 0.05). Intergroup analysis showed no significant differences within each of the group conditions (all *p* > 0.05) (Figure 2D).

#### 3.4.2. LF/HF Ratio

LF/HF measurements using for AUC analysis during the 90 minute period showed no significant time effects between groups (median; 50.9, 25th; 35.3, 75th; 16.3, *p* = 0.704). Analysis of the individual 18 × 5 min time points between groups showed no significant differences between each of the three conditions (all *p* > 0.05). Analysis of same group comparisons showed no significant differences within each of the group conditions (all *p* > 0.05).

## 4. Discussion

The current study implemented a randomised double-blind, placebo-controlled cross over design to investigate the effects of L-THE incorporated into a whey protein-based mango sorbet as a potential functional food product. To our knowledge, this study is the first to examine the effect of a functional food product containing L-THE to determine its physiological effectiveness using a continuous set of BP and HRV measurements. 

The observed results for the ms-L-THE did not produce any significant physiological changes for the parameters of systolic and diastolic BP across the entire 90 minute testing period in comparison to the ms or baseline measurements. Similar to other studies where external factors such as stress or caffeine are used to raise BP, our intervention was designed to cause fluctuations in BP, HR, and HRV due to the carbohydrate response attributed to sorbet consumption [34]. Despite showing initial significant interactions, the systolic/diastolic ratio was a good indicator of the BP effect of both sorbets for within-subject effects. This indicated that consumption of both ms and ms-L-THE caused a postprandial response, in turn, causing the systolic/diastolic to show significant differences as supported by our analysis. Pharmacokinetic clinical data [35] that involved the ingestion of pure L-THE (25−200 mg) suggest peaks in physiological and blood plasma effects occur between 32 and 50 min post oral ingestion (in a fasted state) [29]. Although a similar trend is observed in our current study in terms of a time effect relationship for systolic BP reduction (Figure 2), our study design did not demonstrate any statistically significant physiological effects in comparison to the reported pharmokinetic trends that L-THE exhibits.

Further, as blood plasma concentrations were not studied in our trial, we can only speculate that the observed physiological trends in the current study are in line with the mentioned pharmacokinetic studies [29,35]. The same 200 mg L-THE dosage is clinically reported to acutely attenuate BP (the rise seen in high-stress-response adults), as well as reducing anxiety after stress loading tasks [13]. Additionally, the findings of a relatively recent study have indicated that consumption of L-THE (200 mg) has potential to promote mental health by providing better quality sleep and improvement in cognitive functioning by improving verbal fluency and executive functioning scores [14]. Similarly, a study measuring BP [36] at time points of 20 and 70 min respectively post 97 mg L-THE and 40 mg caffeine intake, highlighted the attenuative effects L-THE has on caffeine. However, this study was limited by reference of only two-time points [36]. Considering previously reported evidence regarding L-THE concentration in the blood post-consumption, it is demonstrated that L-THE potentially antagonises the BP effect of caffeine on participants, without affecting the alertness or mood aspects as well as slowing overall reaction time [37]. In the same study, the authors did not state whether a fasted state was implemented, whilst in comparison, the present study did not implement a cognitive task to raise BP and may in future benefit from an external stimulus to observe the BP effects of L-THE within the food matrix [37].

The initial results suggested that HR was significantly affected using within-subject contrasts however, further analysis revealed no significant interactions. It can be proposed that consumption of both the mango sorbet interventions stabilised HR in contrast to the significant results observed solely in the baseline clinic, however further testing by comparing L-THE in its pure form against L-THE within the food matrix must be conducted to confirm this. As such, these results fall in line with the study by Dodd et al. [10], who also reported no significant differences in HR when pure L-THE was consumed via capsule intake (50 mg); however, this may have occurred due to the low dose provided to participants. In comparison, our results were not on par with those reported by Kimura et al. [15], where consumption of 200 mg L-THE dissolved in water actively resulted in the reduction of HR in response to an acute mental arithmetic stressor task. In the design of our current study, we did not implement a mental stressor task to alter HR, and in future, may benefit from adding a mentally stressing intervention such as arithmetic task. Based on this, it is safe to say that our study is the first to identify that L-THE does not have any effect on HR when consumed within a functional food product and in the absence of environmental stressors; however, from the literature, 200 mg of pure L-THE may acutely lower HR in response to stressful situations which is thought to reflect attenuation of sympathetic nervous activation [15]. Our results do not indicate a potential for L-THE to buffer HRV physiological markers for both HF and LF/HF ratio in the periods of post-consumption. Nonetheless, the presents study suggests that L-THE did reduce the variance of HF outcomes; however, this was not shown to be statistically significant.

In humans, understanding of the physiological responses attributed to L-THE intake where pure L-THE is consumed alongside everyday foods or integrated as part of a food matrix has only been partially investigated to date. Currently, only two known studies aimed at investigating the effect of L-THE consumption embedded within food have been reported [38,39]. The first investigated a nutrient-based drink containing 200 mg of L-THE as well as alpha glycerylphosphorylcholine (α GPC; 25 mg), phosphatidylserine (1 mg), and micronised chamomile (10 mg), however, no BP or HR data was recorded and this particular study reported a decrease in salivary cortisol that supports the potential anti-stress effects of L-THE [39]. The latter study [39] displayed sympathomimetic inhibitory responses post-consumption of 128 mg L-THE within a cacao (60% *w*/*w*) chocolate that resulted in a decrease in BP associated with the L-THE condition. It has been previously suggested by Kakuda et al. [40] that the L-THE mechanism of action relies on the reduction of glutamate release from pre-synapse to the synaptic cleft by acting as an inhibitor of glutamine re-uptake. Additionally, glutamine was also associated with the replenishment of the neurotransmitter pool. This, in turn, inhibits extracellular glutamine uptake by neurons, and its conversion to glutamate via glutaminase enzyme [41]. The recent review by Yoneda et al [42] proposes a possible novel in vitro neurogenic role of L-THE for brain wellness via increase in neurogenesis. Interestingly, the findings of an animal trial using a stress-sensitive strain of mice (senescence-accelerated mice prone 10) that were exposed to stress, had decreased brain volume. However, the same strain of mice that ingested L-THE (6 mg/kg), under the same experimental conditions had a suppression in brain atrophy [43]. Although this is reported to occur based on common neuroprotective outcomes related to stress [42,43], this mechanism of action and the outcomes specific to glutamate biomarkers assessed in the Kakuda et al. [44] study were not assessed in the present study. As there were no statistically significant interactions between the baseline, ms, and ms-L-THE variables, one of the potential mechanisms that may have affected the outcomes was that the L-THE may have bound to the food matrix of the ingredients used in the sorbet (WPC, sucrose, or the mango pulp). Whilst our previous findings indicate that high-performance liquid chromatography (HPLC) analysis of the L-THE extraction from mango pulp yielded a 98% extraction (unpublished data); thus, a potential rational for the integration with mangoes, the potential for L-THE binding to the WPC or sucrose cannot be overlooked. Despite this, it is equally important to acknowledge that HPLC analysis of L-THE is unlikely to reflect the biological outcomes of L-THE in the gastrointestinal tract. Furthermore, it is difficult to determine all known bioactive constituents are present in the mango pulp as well as the WPC and sucrose, which all may affect the kinetical aspects of L-THE including but not limited to: absorption, distribution, elimination, and biotransformation. It is essential to acknowledge the unaccounted bioactive ingredients found within the mango sorbet may have lessened the effects of L-THE, however, to our knowledge, no literature suggest L-THE interacts with WPC or sucrose. Therefore, a similar study design that investigates the effects of L-THE as a mono supplement against other food matrixes is required to determine the physiological outcomes, as well as implementing techniques to monitor L-THE in the circulation as a point of reference for its bioavailability. Based on previous literature, we propose that from the results of this study, L-THE in its pure encapsulated form appears to serve as a medium for consumption to produce its known effects rather than when combined within the food matrix of mango sorbet.

### Limitations and Future Directions

The lack of significant interactions observed in this study can potentially be attributed to a sample size that was reflective of the nature of this study being a pilot trial. Furthermore, due to the exclusion of data (participant exclusion), future studies should account for including a higher number of participants as well as participants with pre existing health conditions such as anxiety and high blood pressure. Furthermore, it is equally important to compare the differences between L-THE in its relatively pure form against L-THE as an integrated component of the food matrix. Although the population was male-only, we can only assume that the results are reflective of the current population included. 

Further, L-THE consumed orally as a component of mango sorbet (200 mg) is a lower dose compared to the pharmacodynamic rat studies by Yokogoshi et al. [45] as well as Yokogoshi and Kobayashi [46], which were both similar in design (1500 mg/day and 2000 mg/kg of L-THE administered intraperitoneally). In future studies, administering a standardised dose adjusted for the kilograms of body weight (kg/bw) is one potential way to maximise the effects of L-THE and determine the ‘ideal’ and personalised dosage for healthy humans as well as potentially increasing the bioavailability of L-THE in the body. However, it is equally important to acknowledge that further research must be conducted where L-THE as a component of a functional food product is compared alongside L-THE in its pure encapsulated form to warrant any clinically relevant claims.

## 5. Conclusions

Our results suggest that consumption of L-THE within the functional food product mango sorbet did not cause significant changes in the physiological responses such as blood pressure and heart rate, as well as parasympathetic nervous system activation (as determined by HF and LF/HF band HRV markers). It is also important to note that reduction of physiological responses via pharmacological interventions does not necessarily mean that the capacity to cope and manage stress is increased. Further food consumption studies with longer duration of consumption should be conducted to evaluate the use of L-THE as a supplement when consumed alongside other foods. It is anticipated that the results of this study will provide baseline information in understanding the activities of the human physiology and its effect on the stress response as well as the important notion to consider the effect food of matrixes have when constructing functional food ingredients.

## Figures and Tables

**Table 1:** Nutrient profile of the mango sorbet with L-Theanine per single serve (100 g).

Average Quantity	Per Serve (100 g)
Energy (kJ)	587
Protein (g)	8.33
Total fat (g)	<1
**SATURATED** (g)	<1
Carbohydrate (g)	28.07
**SUGARS** (g)	26.1
Fibre (g)	1.2
Sodium (mg)	15.55
L-Theanine (mg)	200

The total nutrient profile for the mango sorbet containing the active ingredient L-Theanine (L-THE) expressed as a per 100 g serve. note the mango sorbet (ms) did not contain 200 mg of L-THE.

**Table 2:** The frequencies and Area Under the Curve (AUC) of the visits for baseline, ms-L-THE, and ms sorbets consumption.

Parameters	Baseline Median (1st, 3rd Quartile) Total AUC (/90 min)	ms-L-THE Median (1st, 3rd Quartile) Total AUC (/90 min)	ms Median (1st, 3rd Quartile) Total AUC (/90 min)	*p*-Values			
				Kruskal–Wallis	Friedman		
				All Visits	Baseline	ms-L-THE	ms
**Systolic BP (mmHg)**	137 (123, 151)	133 (123, 143)	137 (123, 148)	0.839	0.147	0.853	0.990
**Total AUC (mmHg/90min)**	26776	26160	27043				
**Diastolic BP (mmHg)**	72 (601, 88)	72 (66,80)	73 (62, 82)	0.922	0.515	0.120	0.491
**Total AUC (mmHg/90min)**	14717	14339	14693				
**Systolic/Diastolic**	32.9 (29.4, 34.95)	31.8 (31.5, 35.7)	34.4 (30.1, 36.9)	0.970	0.761	0.003 *	0.003 *
**Total AUC (ratio/90min)**	33.5	33.2	36.0				
**Heart Rate (bpm)**	74 (66, 77)	73, (68, 77)	73 (67, 77)	0.996	0.003 *	0.066	0.060
**Total AUC (bpm/90min)**	14408	14413	14332				
**HF (Ln) HRV (Hz)**	6.22 (5.20, 6.95)	6.07 (5.27, 7.16)	6.38 (5.24, 7.22)	0.974	0.064	0.534	0.971
**Total AUC (Hz/90min)**	139094	153344	146457				
**LF/HF**	2.96 (2.21, 7.87)	2.55 (2.06, 6.98)	2.60 (1.77, 5.94)	0.704	0.458	0.082	0.330
**Total AUC (ratio/90min)**	1025	905	1054				

The non-parametric frequencies median, 1st and 3rd quartiles and total AUC (expressed as a sum of values from the 0−90 minute period) values for systolic and diastolic BP, systolic/diastolic BP, HR, high-frequency HRV, and LF/HF HRV ratio for the 11 included participants. * Indicates significant *p* < 0.05.
